# Methodological issues of the central mechanism of two classic acupuncture manipulations based on fNIRS: suggestions for a pilot study

**DOI:** 10.3389/fnhum.2022.1103872

**Published:** 2023-02-24

**Authors:** Yuzhu Qu, Jingya Cao, Li Chen, Jing Guo, Zilei Tian, Tianyu Liu, Yulai Gong, Jing Xiong, Zhenfang Lin, Xin Yang, Tao Yin, Fang Zeng

**Affiliations:** ^1^Acupuncture and Tuina School, Chengdu University of Traditional Chinese Medicine, Chengdu, Sichuan, China; ^2^Acupuncture and Brain Science Research Center, Chengdu University of Traditional Chinese Medicine, Chengdu, Sichuan, China; ^3^Sport and Healthy School, Chengdu University of Traditional Chinese Medicine, Chengdu, Sichuan, China; ^4^Department of Neurology, Sichuan Provincial Rehabilitation Hospital, Chengdu, Sichuan, China; ^5^Department of Rehabilitation Medicine, West China Hospital, Sichuan University, Chengdu, Sichuan, China; ^6^Health and Rehabilitation School, Chengdu University of Traditional Chinese Medicine, Chengdu, Sichuan, China

**Keywords:** acupuncture, compound acupuncture reinforcing-reducing manipulation, methodological issues, fNIRS, suggestions

## Abstract

**Background:** Acupuncture reinforcing-reducing manipulation (ARRM) is a necessary procedure of traditional Chinese acupuncture and an essential factor affecting the therapeutic effect of acupuncture. Shaoshanhuo reinforcing method (SSH) and Toutianliang reducing method (TTL) are the most representative ARRMs. They integrate six single ARRMs and pose distinguished therapeutic effects of acupuncture. However, due to the complexity, diversity, and variation, investigating the mechanism of these two classic manipulations is insufficient. The neuroimaging technique is an important method to explore the central mechanism of SSH and TTL. This study attempted to design a randomized crossover trial based on functional near-infrared spectroscopy (fNIRS) to explore the mechanism of SSH and TTL, meanwhile, provide valuable methodological references for future studies.

**Methods:** A total of 30 healthy subjects were finally included and analyzed in this study. fNIRS examination was performed to record the neural responses during the two most representative ARRMs. The cortical activation and the inter-network functional connectivity (FC) were explored.

**Results:** The results found that SSH and TTL could elicit significant cerebral responses, respectively, but there was no difference between them.

**Conclusion:** Neuroimaging techniques with a higher spatiotemporal resolution, combinations of therapeutic effects, and strict quality control are important to neuroimaging studies on SSH and TTL.

## Introduction

Acupuncture reinforcing-reducing manipulation (ARRM) is a necessary procedure of traditional Chinese acupuncture. ARRM is also an essential factor affecting the therapeutic effect of acupuncture. Clinically, acupuncture reinforcing manipulation is suitable for patients with deficiency symptoms (Xu, 2008; Zhang et al., 2019), and acupuncture reducing manipulation is applicable for excess symptoms (Liu, 2006; Li et al., 2016). The appropriate use of ARRMs can enhance the therapeutic effect (Shi, 2021), while adverse use of ARRMs makes the condition worse (Lu et al., 2017a). For instance, compared to regular acupuncture, studies found that acupuncture with reinforcing manipulation could enhance the acupuncture effect on Bell’s palsy with the deficiency of *qi* and blood at the restoration stage (Wang et al., 2008). However, the underlying mechanism of ARRMs is poorly understood.

In acupuncture practice, central integration plays an important role in achieving therapeutic effects (Yoo et al., 2004; Cai et al., 2018; Yu et al., 2018). The application of neuroimaging techniques to elucidate acupuncture’s central mechanisms has become a hot research direction in acupuncture field (Zhang et al., 2020a). While, there was no acupuncture neuroimaging study on ARRMs over the past 20 years (Li et al., 2003; Lu et al., 2017b; Yu et al., 2019; Si et al., 2021). What are the reasons for the conflict between the necessary procedure of acupuncture and the absence of its mechanism research? Acupuncture neuroimaging studies mainly focus on the results of acupuncture. ARRMs neuroimaging studies not only focus on the results but also pay more attention to the process of changes. Besides, ARRMs are known for complex and variable manipulations, long manipulation time, large stimulation, and intense needle sensations on individuals. To investigate the mechanism of ARRMs, neuroimaging techniques require excellent spatiotemporal resolution and capacity for real-time monitoring in natural clinical environment.

Among various ARRMs, the reinforcing manipulation (Shaoshanhuo, SSH) and the reducing manipulation (Toutianliang, TTL) are the most representative ones. They integrate six frequently used single ARRMs, so that they can produce much more significant responses in individuals (Zhang et al., 2020b). In clinical practice, SSH and TTL have distinguished the therapeutic effects of acupuncture. SSH has special effects for extreme deficiency (Wang, 2005) and cold symptoms (Yuan et al., 2017), and TTL produces special effects for severe excessive (Liu and Ma, 2014) and heat symptoms (Yang, 1988). Further, the acupoint is the basic part of SSH and TTL manipulations. *Quchi* (LI 11) is one of the most frequently used acupoints in experimental studies (Chavez et al., 2017). LI 11 is the “he-acupoint” of the large intestine meridian, where abundant meridian *qi* converges. It is located around the elbow joint, where the muscles are thick with many peripheral nerves, making it safe and easy to obtain needle sensation. Therefore, performing SSH and TTL on LI 11 is a good choice for ARRMs neuroimaging research. In recent years, functional near-infrared spectroscopy (fNIRS) has emerged as a well-established imaging tool for neuroscience research (Eastmond et al., 2022). It has a high temporal resolution (Tak and Ye, 2014) and the capacity for monitoring in real clinical settings (Dybvik and Steinert, 2021; Gossé et al., 2022). fNIRS has gained increasing attention and application in acupuncture research (Fernandez Rojas et al., 2019; Ghafoor et al., 2019; Wong et al., 2021). Therefore, fNIRS may offer the possibility to elucidate the central mechanism of SSH and TTL.

Based on the above conception, we designed a randomized crossover trial to investigate SSH and TTL by using fNIRS. The aims were to explore the mechanism of these two classic ARRMs, meanwhile, to provide methodological references for future studies on the mechanism of SSH and TTL based on neuroimaging techniques.

## Materials and method

### Participants

Thirty-three eligible participants were recruited at Chengdu University of Traditional Chinese Medicine from 2 October 2021 to September 2022. All eligible participants met the inclusion criteria and provided written informed consent before participation.

#### Inclusion criteria

Participants who met all the following criteria were included: (1) male and right-handed; (2) aged between 18 and 30 years; (3) college degree or above; (4) without organic or functional diseases or psychological disorders; and (5) not participating in other clinical trials currently.

#### Exclusion criteria

Participants who met any of the following criteria were excluded: (1) having skin lesions around the stimulated acupoint (left *Quchi*); (2) having any contraindications of acupuncture, such as fear of acupuncture, needle fainting, intolerance to needle sensation, etc.; and (3) whose Self-rating Anxiety Scale (SAS; Dunstan et al., 2017) or Self-rating Depression Scale (SDS) scores ≥50 (Dunstan et al., 2017).

### Acupuncture manipulation

**Figure 1:**
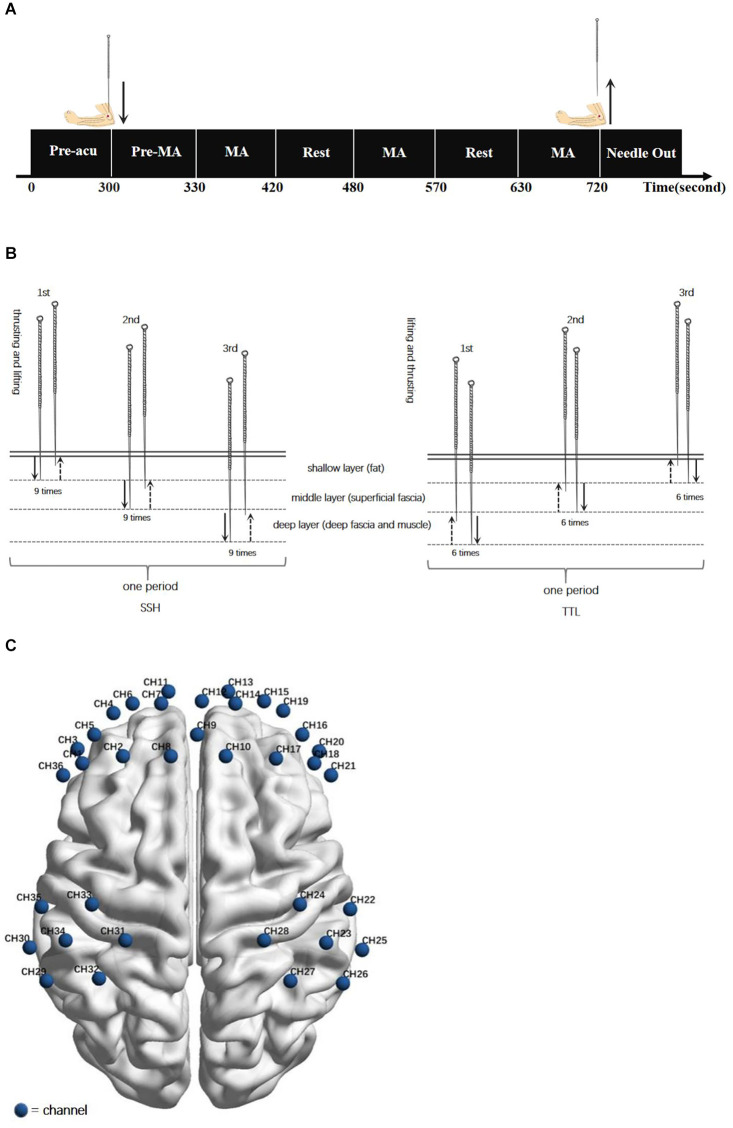
**(A)** Acupuncture experiment paradigm. The experiment included a 300-s resting-state scanning (Pre-acu rest), a 30-s pre-manipulation (Pre-MA) stage for inserting a needle and twirling-rotating needle to obtain *deqi* sensation, and three 90-s blocks of acupuncture manipulation (MA) following by a 60-s interval resting state. **(B)** Acupuncture manipulations: SSH was from the shallow to the deep with nine times heavy swift thrusting and gentle slow lifting at each layer for one period, repeating the period for three times. TTL was from the deep to the shallow with six times heavy swift lifting and gentle slow thrusting at each layer for one period, repeating the period for three times. **(C)** fNIRS channel configuration. Locations of channels in the sensorimotor cortex and prefrontal cortex during the task fNIRS scan.

To avoid the influence of individual differences, a randomized crossover protocol was used in this study. According to previous acupuncture neuroimage studies (Bai et al., 2009; Rheu et al., 2011; Claunch et al., 2012), participants received SSH and TTL on separate days, with a washout of 1 day. The order of acupuncture manipulation was determined by the computer-generated random number table. All acupuncture manipulations were performed by one licensed acupuncturist with clinical experience of over 5 years. SSH and TTL were performed at *Quchi* (LI11, left side) using the disposable needle (size 0.25 × 40 mm, *Hwatuo*, Suzhou Medical Supplies Factory Co., Ltd., Suzhou, China). The acupuncture experiment paradigm was shown in Figure 1A. The details of the following acupuncture manipulations were shown in Figure 1B.

The depth of needling is divided into three layers, shallow, middle, and deep, corresponding to the subcutaneous layer (fat), loose connective tissue layer (superficial fascia), and dense connective tissue (deep fascia and muscle; Chen, 2017).

#### SSH

The participant was asked to inhale through the nose and exhale through the mouth normally. During the participant’s exhalation, inserted the needle into the shallow layer. After obtaining *deqi* sensation, conducted heavy swift thrusting and gentle slow lifting nine times, always remaining within the shallow layer. Then insert the needle more deeply into the middle layer, and keeping the needle at this level, conducted heavy swift thrusting and gentle slow lifting nine times. Then, penetrated the needle into the deep layer and conducted heavy swift thrusting and gentle slow lifting nine times within this deep level. The period of the above manipulation is 90 s. Subsequently, withdrew the needle to the shallow layer, and repeated the above period three times with an interval of 60 s. Then on the participant’s inhalation quickly withdrew the needle.

#### TTL

The participant was asked to inhale through the nose and exhale through the mouth normally. During the participant’s inhalation, inserted the needle into the deep layer. After obtaining *deqi* sensation, conducted heavy swift lifting and gentle slow thrusting six times, always remaining within the deep layer. Then withdrew the needle shallower to the middle layer, and keeping the needle at this level, conducted heavy swift lifting and gentle slow thrusting six times. Then, withdrew the needle to the shallow layer and conducted heavy swift lifting and gentle slow thrusting six times within this shallow level. The period of the above manipulation is 90 s. After that, penetrated the needle into the deep layer, and repeated the above period three times with an interval of 60 s. Finally, on the participant’s exhalation slowly removed the needle.

### Clinical metrics assessments

#### Cognitive assessments

At the baseline, the mindful attention awareness scale (MAAS; Black et al., 2012) was measured to ensure that participants could keep sufficient attention and awareness during acupuncture stimulation.

#### Needle sensation evaluations

After each acupuncture stimulation, the Chinese version of the Modified Massachusetts General Hospital Acupuncture Sensation Scale (C-MMASS; Kong et al., 2007; Yu et al., 2012) was used to evaluate the needle sensation. we further used the 10-point Visual Analogue Scale to measure the heat and cool needle sensations of SSH and TTL.

### fNIRS data acquisition

The fNIRS scan lasted for 720 s (Figure 1C). During the scan, participants were asked to close their eyes and keep relaxed without falling asleep. A non-invasive multi-channel continuous wave fNIRS instrument (NIRx Medical Technologies, New York) was utilized to record the oxyhemoglobin (oxy-Hb) and deoxyhemoglobin (deoxy-Hb) signals. The absorption of near-infrared light was measured at the wavelengths of 785 and 830 nm and the sampling frequency was 3.91 Hz. Same as our recent study (Chen et al., 2022), the sensorimotor cortex (SMC) and prefrontal cortex (PFC) was selected as regions of interest (ROI). As shown in Figure 1C, two 2 × 3 optode probe sets consisting of seven channels (CHs) were placed at the bilateral SMC, and one 3 × 5 optode probe set consisting of 22 CHs was placed at the bilateral PFC. The fNIRS optode was placed according to the international 10–10 EEG system (Tsuzuki et al., 2017), with Fpz and Cz as reference points. The separation distance of sources and detectors was set to 2.5–3 cm. The corresponding anatomical positions of fNIRS CHs were measured by an electromagnetic 3D digitizer system (PATRIOT; Polhemus) and registered on the Montreal Neurological Institute MNI brain space using a virtual registration method (Supplementary Table S1).

### fNIRS data analysis

#### fNIRS data preprocessing

fNIRS data were processed by the Homer2 software package^1^ based on MATLAB (R2013b, MathWorks, Inc., Natick, MA, USA). Prior to data preprocessing, the fNIRS signals were visually quality-checked for motion artifacts. The preprocessing process included five steps: (1) the raw NIRS light intensity was converted to an optical density (OD) signal; (2) identified, removed, or re-calibrated channels with bad signals and long periods of motion artifacts by the function *hmr Motion Artifact By Channel*; (3) motion correction was conducted by the function *hmr Motion correction Spline* (input parameters: *p* = 0.99, turnon = 1); (4) removed physiological noises by a 0.01–0.2 Hz bandpass filter according to previous fNIRS based studies (Scholkmann et al., 2014; Pinti et al., 2018; Si et al., 2021); and (5) converted the filtered OD data into oxy-Hb, deoxy-Hb, and total-Hb (defined as the sum of oxy-Hb and deoxy-Hb) by the modified Beer-Lambert law (Delpy et al., 1988).

#### Cortical activation analysis

We used the oxy-Hb signal to characterize the functional activity of ROI since it has a better signal-to-noise ratio (SNR) and is more reliable and sensitive than deoxy-Hb signal (Strangman et al., 2002; Sato et al., 2004; Fernandez Rojas et al., 2017). Cortical activation analysis for each subject and the group-level were obtained based on a general linear model (GLM), by means of the NIRS-SPM software package^2^, (Ye et al., 2009) on the MATLAB platform. In NIRS-SPM, a design matrix was created, composed of the task-related regressor modeling the hemodynamic response to the simulated block-design experiment, plus the constant term [design parameters: Hemodynamic basic functions: hrf (with time and dispersion derivatives); Detrending: Wavelet-MDL; Low-pass filter: hrf; Correct for serial correlations: none.; Jang et al., 2009]. The regression coefficient (β-values) was calculated through the least square estimation for each channel in each subject. Then β-values for all subjects were used to run statistical analyses at group-level.

#### Functional connectivity analysis

To reflect the functional connectivity patterns of ROI, we calculated the *Pearson* correlation coefficients between two of the 36 CHs based on oxy-Hb signals and subsequently converted the *r* values into *z* values using Fisher’s z transformation method (Ghafoor et al., 2019) to generate FC matrices for each subject at the resting state, SSH state, and TTL state.

### Statistical analysis

The statistical analysis consists of two parts: the within-group comparison of SSH/TTL and resting period and the between-group comparison of SSH and TTL. The within-group comparison was conducted with paired *t*-test. The between-group comparison was conducted with two sampled *t*-test based on the delta value (Δ) of cortical activity and FC (Δ = acupuncture manipulation − pre-acupuncture rest). The significance threshold was set to *p* < 0.05. To minimize the risk of type I errors, the *p*-values were corrected with false discovery rate (FDR) correction for multiple comparisons.

## Results

### Demographics and clinical metrics

Thirty-three subjects completed SSH and TTL manipulations. Four subjects in SSH and three subjects in TTL were excluded due to poor data quality. There were no significant differences between SSH and TTL in all metrics (Supplementary Table S2).

### Cortical activation and FC in acupuncture manipulation

**Figure 2:**
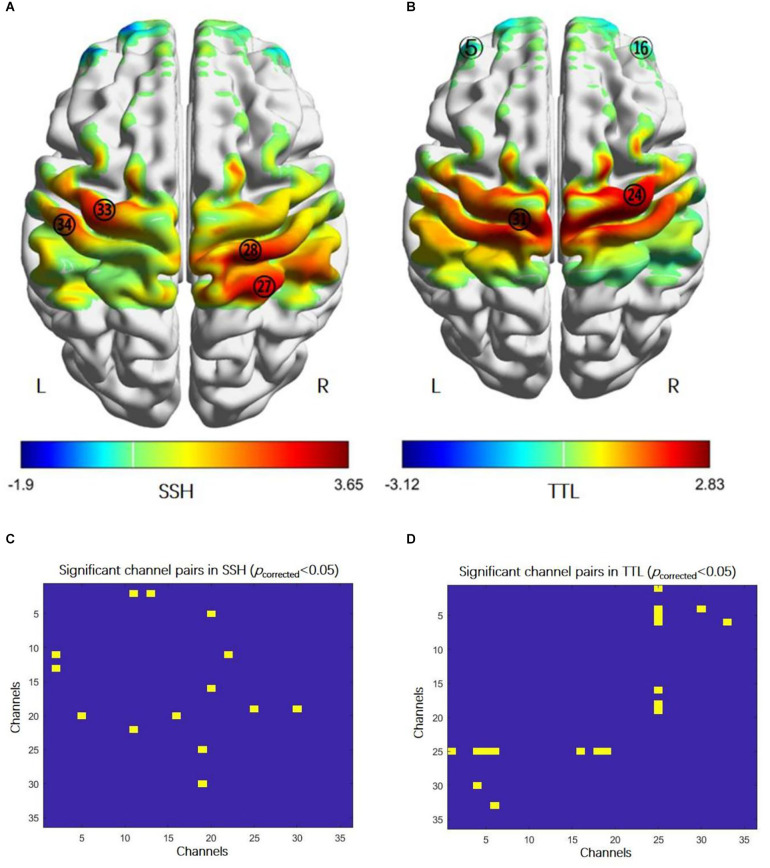
Cortical activation and functional connectivity in acupuncture manipulation. **(A)** The activation regions: the bilateral primary motor cortex (MI, CH28 and CH33), the right somatosensory association cortex (CH27), and the left primary somatosensory cortex (CH34) (paired *t*-test, *p* < 0.05, FDR correction). **(B)** The activation regions: the bilateral prefrontal cortex (PFC, CH5, and CH16) and the bilateral MI (CH24 and CH31) (paired *t*-test, *p* < 0.05, FDR correction). **(C)** FC enhanced between: the right and left prefrontal cortex (PFC, CH5-CH20, and CH16-CH20), the right PFC (CH2-CH11, CH2-CH13), the left PFC and the right primary somatosensory cortex (CH11-CH22), the right PFC and the right primary somatosensory cortex (CH19-25), and the right PFC and the left primary somatosensory cortex (CH19-CH30) (paired *t*-test, *p* < 0.05, FDR correction). **(D)** Higher FC between: the left PFC and the right primary somatosensory cortex (CH1-CH25, CH4-CH25, CH5-CH25, and CH6-CH25), right PFC and the right primary somatosensory cortex (CH16-CH25, CH18-CH25, and CH19-CH25), the left PFC and the left primary somatosensory cortex (CH4-CH30), and the left PFC and the left primary motor cortex (CH6-CH33) (paired *t*-test, *p* < 0.05, FDR correction). Abbreviations: FC, functional connectivity; SSH, Shaoshanhuo reinforcing method; TTL, Toutinaliang reducing method.

In SSH, the bilateral primary motor cortex (MI), the left primary somatosensory cortex (SI), and the right somatosensory association cortex were activated (Figure 2A, paired *t*-test, *p* < 0.05, FDR correction); the FC between the right and left PFC,

The PFC and SI (Figure 2C, paired *t*-test, *p* < 0.05, FDR correction). In TTL, the bilateral PFC and the bilateral MI were activated (Figure 2B, paired *t*-test, *p* < 0.05, FDR correction); the FC between the PFC and SI, the left PFC and left MI were increased (Figure 2D, paired *t*-test, *p* < 0.05, FDR correction). However, there was no difference in cortical activation and FC between SSH and TTL under the corrected threshold.

## Discussion

This study was the first time to explore the central mechanism of SSH and TTL based on neuroimaging techniques, and the first time to investigate potential methodological issues of these two classic ARRMs neuroimaging studies. The results found that SSH and TTL could induce cortical responses, respectively, but there was no difference between them. Based on existing research and this study, we may have the following methodological suggestions.

### Neuroimaging techniques with higher spatiotemporal resolution may be effective for exploring the mechanism of SSH and TTL

Due to its complexity, diversity, and variation, investigating the central mechanism of SSH and TTL requires neuroimaging techniques with high spatiotemporal resolution. On the one hand, the changes elicited are extremely subtle. Neuroimaging techniques with excellent temporal resolution are sufficient to catch these subtle changes over time. On the other hand, studies have revealed that activations induced by acupuncture are mainly in deep brain tissues (Huang et al., 2012). Neuroimaging techniques with high spatial resolution are necessary to record changes induced by SSH and TTL.

**Figure 3:**
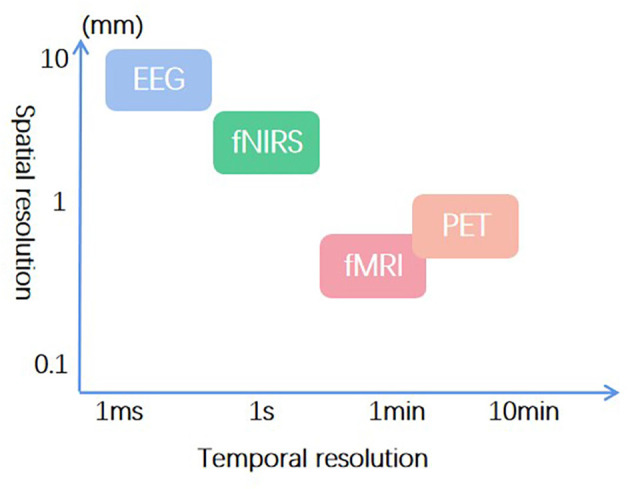
Temporal and spatial resolution of frequently used neuroimaging techniques in acupuncture research.

Among acupuncture neuroimaging studies, electroencephalogram (EEG), fNIRS, functional magnetic resonance imaging (fMRI), and positron emission tomography (PET) are most frequently used. EEG provides the best temporal resolution (Cao et al., 2021), and fMRI has the optimal spatial resolution (Chou et al., 2020). Considering the temporal resolution, spatial resolution, and feasibility in a real clinical condition, fNIRS was used in this study (Figure 3). Its temporal resolution is next only to EEG (Li et al., 2022), and the spatial resolution is second to fMRI (Chen et al., 2020). Moreover, fNIRS has excellent ecology validity for use in real clinical settings. Unfortunately, this study encountered some limitations in utilizing fNIRS for SSH and TTL research. Consequently, combing higher temporal and spatial resolution is recommended for future neuroimaging studies on SSH and TTL.

Currently, fMRI-EEG can achieve the optimal spatiotemporal resolution (He and Liu, 2008). EEG-fNIRS can improve temporal resolution and feasibility in real clinical settings (Uchitel et al., 2021). At the same time, applications of multimodal neuroimaging may bring potential difficulties in the study design of SSH and TTL, such as the judgment of the needle sensation, acupuncture manipulations on the upper body, and the compatibility between neuroimaging techniques. Nevertheless, neuroimaging techniques with higher spatiotemporal resolution are still more applicable for investigating the central mechanism of SSH and TTL.

### Combing with therapeutic effects may be more appropriate to elucidate the central mechanism of SSH and TTL

SSH and TTL play an important role in affecting acupuncture’s therapeutic effects. To understand SSH and TTL in depth, the relationship between these two manipulations and their therapeutic effects should be considered sufficiently. Though using healthy subjects in the research can receive good compliance, it is difficult to characterize the effects of ARRMs on treating diseases. Thus, there is a growing trend in acupuncture neuroimaging studies to change subjects from healthy subjects to patients (Cai et al., 2018; Zhang et al., 2021). Research showed that positive results were reported in acupuncture studies on patients, while negative results were found on healthy subjects only (Qiu et al., 2020). Health subjects might be the possible factor for no FC difference between SSH and TTL. Hence, it is better to use diseases, combing the therapeutic effects to investigate the mechanism of SSH and TTL. SSH can be used for treating conditions with deficiency and cold symptoms, and TTL can be selected for controlling disorders with excessive and heat symptoms. For example, rheumatoid arthritis with cold and heat types can be applied as a disease carrier for exploring SSH and TTL. Therefore, it is suggested that future studies combine with therapeutic effects to investigate the central mechanism of SSH and TTL.

### Strict and detailed quality control is indispensable in research of SSH and TTL

Strict and detailed quality control is the guarantee of acquiring reliable results. In this study, rigorous quality control was performed from three aspects: participants, practitioners, and the research environment. For participants, all healthy subjects were recruited in accordance with the inclusion criteria. They were required to maintain a regular lifestyle, away from alcohol and caffeine for 24 h before fNIRS scans. Then they were asked to sit quietly with eyes closed, not thinking about specific things, and concentrate on the experience of needle sensations during acupuncture task scans. For practitioners, this study was conducted following the pre-established standard operating procedure (SOP). All acupuncture manipulations were completed by one trained acupuncturist. For the research environment, it was kept quiet and dark to avoid the interference of light and sound on fNIRS signals. All scans were carried out by the same fNIRS equipment.

For future studies, it is recommended to perform strict and detailed quality control. It requires subjects’ good compliance, including focusing on feeling the needle sensation during acupuncture task scans and complying with trial requirements. SOP should be made and practitioners should be trained in advance. Finally, the research environment is needed to avoid contraindications of neuroimaging techniques’ scans and manipulations of SSH and TTL.

## Conclusion

As the first neuroimaging study on SSH and TTL, this study found that these two manipulations could elicit significant cerebral responses, but there was no difference between them. To further explore the central mechanism of SSH and TTL, it is recommended to apply neuroimaging techniques with a higher spatiotemporal resolution, combine with therapeutic effects, and perform strict quality control.

## Data availability statement

The original contributions presented in the study are included in the article/Supplementary material, further inquiries can be directed to the corresponding author/s.

## Ethics statement

The studies involving human participants were reviewed and approved by the Institutional Review Boards and Ethics Committees of Chengdu University of Traditional Chinese Medicine (No.: 2021QKL-001). It was registered at the Chinese Clinical Trial Registry (http://www.chictr.org.cn, No.: ChiCTR2100051886). The patients/participants provided their written informed consent to participate in this study.

## Author contributions

FZ and TY were responsible for this study. FZ, TY, YQ, JC, and LC contributed to the study conception and design. JG, TL, YG, and JX participated in material preparation and participants recruitment. XY and ZL contributed to participant recruitment. YQ and ZT performed the data collection and data analysis. YQ wrote the first draft of the manuscript. All authors contributed to the article and approved the submitted version.

## Footnotes

^1^ http://homer-fnirs.org/

^2^ https://www.nitrc.org/frs/?group_id=621

## Abbreviations

FC: Functional connectivity
SSH: Shaoshanhuo reinforcing method
TTL: Toutinaliang reducing method.
