# Supplementary material for: Methodological issues of the central mechanism of two classic acupuncture manipulations based on fNIRS: suggestions for a pilot study
Source: Front Hum Neurosci. 2023 Feb 24;16:1103872. doi: 10.3389/fnhum.2022.1103872 (PMC9999014; doi:10.3389/fnhum.2022.1103872)
Supplement: Supplementary file 1 [file Table_1.pdf]

Supplementary Table S1 Average coordinates, anatomical regions, and atlas probabilities of channels

**CH**	**MNI coordinates**			**Anatomical Region**	**Probability**
	**X**	**Y**	**Z**		
**1**	-47	43	24	Left Middle Frontal Gyrus-BA45	0.75
**2**	-30	46	42	Left Middle Frontal Gyrus-BA9	0.54
**3**	-49	49	-1	Left Inferior Frontal Gyrus-BA46	0.32
**4**	-34	64	-9	Left Middle Frontal Gyrus-BA11	0.79
**5**	-42	55	15	Left Middle Frontal Gyrus-BA46	0.93
**6**	-26	68	4	Left Superior Frontal Gyrus-BA10	0.76
**7**	-14	68	24	Left Superior Frontal Gyrus-BA10	0.69
**8**	-10	46	52	Left Superior Frontal Gyrus-BA9	0.53
**9**	1	55	41	Left Superior Frontal Gyrus-BA9	0.63
**10**	13	46	53	Right Superior Frontal Gyrus-BA9	0.53
**11**	-11	73	-5	Left Superior Frontal Gyrus-BA11	0.38
**12**	3	69	13	Right Superior Frontal Gyrus-BA10	0.50
**13**	14	73	-5	Right Superior Frontal Gyrus-BA11	0.41
**14**	17	68	25	Right Superior Frontal Gyrus-BA10	0.60
**15**	29	69	5	Right Superior Frontal Gyrus-BA10	0.74
**16**	45	55	16	Right Middle Frontal Gyrus-BA46	1
**17**	34	45	43	Right Middle Frontal Gyrus-BA9	0.81
**18**	50	43	26	Right Middle Frontal Gyrus-BA45	0.79

**19**	37	65	-10	Right Middle Frontal Gyrus-BA11	0.73
**20**	52	48	0	Right Inferior Frontal Gyrus-BA46	0.30
**21**	57	38	1	Right Inferior Frontal Gyrus-BA45	0.62
**22**	65	-18	44	Right Primary Somatosensory Cortex-BA1	0.54
**23**	55	-32	58	Right Primary Somatosensory Cortex-BA1	0.44
**24**	44	-16	68	Right Primary Motor Cortex-BA4	0.59
**25**	70	-35	29	Right Primary Somatosensory Cortex-BA1	0.26
**26**	62	-49	46	Right Supramarginal Gyrus-BA40	0.92
**27**	40	-48	68	Right Somatosensory Association Cortex-BA7	0.39
**28**	29	-31	75	Right Primary Motor Cortex-BA4	0.69
**29**	-62	-48	45	Left Supramarginal Gyrus-BA40	0.96
**30**	-69	-34	27	Left Primary Somatosensory Cortex-BA1	0.34
**31**	-29	-31	74	Left Primary Motor Cortex-BA4	0.72
**32**	-40	-47	68	Left Somatosensory Association Cortex-BA7	0.28
**33**	-43	-16	67	Left Primary Motor Cortex-BA4	0.62
**34**	-54	-31	57	Left Primary Somatosensory Cortex-BA1	0.41
**35**	-64	-17	42	Left Primary Somatosensory Cortex-BA1	0.40
**36**	-55	38	-1	Left Inferior Frontal Gyrus-BA45	0.76
