# Supplementary material for: Methodological issues of the central mechanism of two classic acupuncture manipulations based on fNIRS: suggestions for a pilot study
Source: Front Hum Neurosci. 2023 Feb 24;16:1103872. doi: 10.3389/fnhum.2022.1103872 (PMC9999014; doi:10.3389/fnhum.2022.1103872)
Supplement: Supplementary file 2 [file Table_2.pdf]

Supplementary Table S2 Demographics and clinical metrics

	SSH	TTL	*p*-values
Subjects	29	30	/
Age	20.41±1.94	20.63±2.41	0.702
Gender	Male	Male	/
MAAS	69.14±8.34	67.97±8.27	0.590
CMMASS	1.48±2.14 (Heat sensation)	0.87±1.65 (Cool Sensation)	0.221

Abbreviations: SSH, Shaoshanhuo reinforcing method; TTL, Toutinaliang reducing method; MAAS, the mindful attention awareness scale; C-MMASS, the Chinese version of the Modified Massachusetts General Hospital Acupuncture Sensation Scale.
